# Influence of the Processing Method on the Nano-Mechanical Properties and Porosity of Dental Acrylic Resins Fabricated by Heat-Curing, 3D Printing and Milling Techniques

**DOI:** 10.3390/dj13070311

**Published:** 2025-07-10

**Authors:** Marina Imre, Veaceslav Șaramet, Lucian Toma Ciocan, Vlad-Gabriel Vasilescu, Elena Iuliana Biru, Jana Ghitman, Mihaela Pantea, Alexandra Ripszky, Adriana Lucia Celebidache, Horia Iovu

**Affiliations:** 1 Discipline of Prosthodontics, Faculty of Dentistry, “Carol Davila” University of Medicine and Pharmacy, 37 Dionisie Lupu Street, District 2, 020021 Bucharest, Romania; marina.imre@umfcd.ro (M.I.); mihaela.pantea@umfcd.ro (M.P.); 2 Private Practice, 011841 Bucharest, Romania; veaceslav@saramet.eu (V.Ș.); office@dentexcela3.ro (A.L.C.); 3 Discipline of Technology of Dental Prosthetics, Faculty of Dentistry, “Carol Davila” University of Medicine and Pharmacy, Dionisie Lupu Street, No. 37, District 2, 020021 Bucharest, Romania; 4 Advanced Polymer Materials Group, National University of Science and Technology Politehnica Bucharest, 1–7 Gh. Polizu Street, 011061 Bucharest, Romania; iuliana.biru@upb.ro (E.I.B.); jana.ghitman@upb.ro (J.G.); horia.iovu@upb.ro (H.I.); 5 Discipline of Biochemistry, Faculty of Dentistry, “Carol Davila” University of Medicine and Pharmacy, 8 Eroilor Sanitari Blvd, 050474 Bucharest, Romania; alexandra.ripszky@umfcd.ro

**Keywords:** acrylic resins, computer-aided design, computer-aided manufacturing, 3D printing, nanoindentation, nano-mechanical properties, porosity

## Abstract

**Background**: Acrylic resin-based materials are a versatile category used extensively in various dental applications. Processed by current modern technologies, such as CAD/CAM technologies or 3D printing, these materials have revolutionized the field of dentistry for the efficient creation of dental devices. However, despite their extensive use, a limited number of comparative studies exist that investigate how different processing methods—such as traditional techniques, 3D printing, and CAD/CAM milling—impact the nano-mechanical behavior and internal porosity of these materials, which are critical for their long-term clinical performance. **Objectives:** The purpose of this study is to evaluate the nanomechanical properties (hardness, elasticity, and stiffness) and micro-porosity of acrylic resin-based materials indicated for temporary prosthodontic appliances manufactured by new technologies (milling, 3D printing) compared to traditional methods. **Methods:** The hardness, elasticity, and stiffness measurements were performed by the nano-metric indentation method (nanoindentation), and the quantitative morphological characterization of the porosity of the acrylic resin samples obtained by 3D printing and CAD/CAM milling was performed by micro-computed tomography. **Results:** According to nanomechanical investigations, CAD/CAM milling restorative specimens exhibited the greatest mechanical performances (E~5.233 GPa and H~0.315 GPa), followed by 3D printed samples, while the lowest mechanical properties were registered for the specimen fabricated by the traditional method (E~3.552 GPa, H~0.142 GPa). At the same time, the results of porosity studies (micro-CT) suggested that 3D printed specimens demonstrated a superior degree of porosity (temporary crown—22.93% and splints—8.94%) compared to CAD/CAM milling restorative samples (5.73%). **Conclusions:** The comparative analysis of these results allows for the optimal selection of the processing method in order to ensure the specific requirements of the various clinical applications.

## 1. Introduction

The mechanical and biological characteristics of oral appliances have been greatly enhanced by recent developments in polymeric materials and digital manufacturing technologies, such as additive 3D printing and subtractive CAD/CAM milling, which provide improved clinical performance, precision, and reproducibility [1]. Modern dentistry invokes the evaluation of the quality of materials based on physical, chemical, and mechanical tests to assess their typical properties. Comparative analysis of their characteristics is the basis for the correct choice in a specific application, as a close connection between the clinical success of materials and certain properties has been demonstrated.

Because of their favorable physical and mechanical properties, acrylic resins remain widely used in temporary and permanent dental restorations. Recent studies highlight not only their adequate mechanical strength and biocompatibility but also their adaptability to digital fabrication workflows, including 3D printing and CAD/CAM milling [1,2,3,4]. These materials are employed in the fabrication of temporary crowns, splints, and surgical guides due to their good dimensional stability, aesthetic characteristics, and polymerization control [5,6,7]. Both conventional laboratory techniques and digital processing methods are routinely used in dental practice today, with increasing preference for subtractive and additive technologies that offer improved standardization and reproducibility [8,9,10,11].

Research in the field of dental polymer chemistry has brought improvements in the formula of resins, emphasizing the importance of the organic phase of these materials [12,13]. An adequate molecular structure of organic monomers undergoing polymerization reactions is the first step on the way to a complex dental material with high biocompatibility, antimicrobial capacity, healing and remineralization capacity, adequate aesthetic properties, and good mechanical properties. In the case of direct restorations, nanocomposites based on organic resins such as glycidyl methacrylate-biphenol-A (BisGMA) or bisphenol-A dimethacrylate (BisEMA) are mainly used in combination with other urethane dimethacrylates and inorganic fillers of various sizes and shapes, such as colloidal silicon [14,15] or glass ionomers [16]. These resins offer superior mechanical properties, dimensional stability, and better retention compared to typical composites with microfillers [17,18]. In terms of processing, traditional methods such as manual molding of crowns and bridges have long been the standard in the manufacture of dental prosthodontics applications. These procedures often involve a long time to complete and can be subject to human error. However, traditional techniques remain relevant due to lower upfront costs and familiarity with procedures among practitioners. The advantages brought by the new technologies used in recent decades, such as 3D printing and CAD/CAM milling, have revolutionized the field of dentistry. The integration of advanced manufacturing technologies into dental practice represents a significant evolution, which promises to improve the quality of dental treatments through multiple advantages, including those regarding high precision and optimized personalization of treatments.

CAD/CAM (Computer-Aided Design/Computer-Aided Manufacturing) technology was introduced in dentistry in 1980 and allowed for the production of crowns, bridges, and other prosthetic devices with high precision through subtractive and additive processes. Computer-aided design can transform digital data into accurate final parts and automated manufacturing of dental devices, with the advantage of significantly reducing production time and accurately reproducing dental structures. Three-dimensional printing is based on an additive process, in which the material is added layer by layer to build structures that would be impossible or impractical with traditional technologies [19,20]. This method not only reduces material waste but also allows for the rapid creation of prototypes and complex final parts with fine detail, while also having significant flexibility and design freedom compared to traditional methods. Three-dimensional printing is particularly useful in the production of surgical guides, orthodontic and bruxism splints, as well as temporary crowns. The ability to print biocompatible materials directly from digital designs provides an increased level of customization and adaptation in dental practice. The assimilation and integration of these advanced technologies into dental practice decisively improves clinical outcomes, responding to a major desire, that of behaving optimally to the ever-growing needs of patients [21,22,23,24,25,26,27,28,29].

Alongside modern processing techniques, advanced material characterization methods play a critical role in optimizing the clinical performance of restorative dental materials. Among these, nanoindentation has emerged as one of the most reliable and precise methods for evaluating mechanical properties at the nanoscale, particularly in heterogeneous materials such as polymer-based resins used in prosthodontics. This technique allows for the accurate quantification of parameters like hardness, Young’s modulus, and stiffness, which are essential in assessing how materials behave under functional loading conditions. The ability to perform localized measurements at the micro- and nanoscale is especially valuable for dental polymers, where variations in internal structure and surface morphology can substantially impact clinical outcomes. Simultaneously, porosity remains a key structural feature influencing the long-term performance of dental restorations. Open and closed pores may act as stress concentrators, promoting material fatigue, crack propagation, or fluid infiltration. Parameters such as pore volume, size distribution, and interconnectivity are directly affected by the processing method and can be effectively visualized and quantified using high-resolution micro-computed tomography (micro-CT). This non-destructive imaging technique provides a detailed three-dimensional view of the internal structure, offering valuable insights into the material’s homogeneity and potential weak points. Through the integration of nanoindentation and micro-CT analysis, the current study provides a robust comparative framework for evaluating the nanomechanical behavior and microstructural porosity of PMMA-based materials fabricated via traditional, subtractive, and additive techniques. These insights are intended to support evidence-based selection of materials and fabrication workflows tailored to the specific demands of temporary prosthodontic applications [13,30,31,32,33,34].

The present study was based on the hypothesis that, although modern digital manufacturing techniques such as 3D printing and CAD/CAM milling significantly reduce the time required to fabricate dental restorations, they involve the use of new polymer-based materials whose nanomechanical performance and internal microstructure may differ substantially from those of conventionally processed resins, and whose clinical efficiency remains insufficiently characterized.

## 2. Aim

The aim of this study was to comparatively evaluate the nano-mechanical behavior and internal porosity of dental acrylic resins processed using three different manufacturing techniques: traditional heat-curing, additive manufacturing via 3D printing, and subtractive CAD/CAM milling. Considering the increasing implementation of digital fabrication technologies in clinical prosthodontics, the scope of the investigation was to determine whether the newer polymer-based materials—specifically developed for additive and subtractive workflows—can match or exceed the mechanical performance of conventional resins commonly used in temporary dental restorations. This research was designed to test the hypothesis that, despite offering significant advantages in terms of fabrication time and digital precision, modern processing methods may involve materials whose internal structure and mechanical properties are not yet fully understood or standardized. By analyzing and comparing the elastic modulus, hardness, stiffness, and porosity (including type and distribution of pores), this study sought to provide clinically relevant data that would support the optimal selection of material and processing technique for temporary dental applications based on performance criteria rather than convenience or familiarity. Furthermore, the investigation aimed to highlight the practical implications of material behavior at the micro- and nanoscale level, which may significantly influence the long-term stability, resistance to wear, and dimensional reliability of dental prostheses.

## 3. Materials and Methods

Four PMMA materials differentiated by manufacturing technique have been selected. Materials examined in this study, chosen for testing, were PMMA Superpont C + B (Spofadental, Jičín, Czechia), a traditional heat-cured acrylic resin, PMMA processed by 3D printing for temporary appliances (crowns and splints), and subtractively processed milled PMMA. From each PMMA material, six identical samples were processed using different techniques (12 mm diameter, 3 mm thickness).

### 3.1. Experimental Samples

Samples were prepared in the shape of circular discs (12 mm diameter and 3 mm thickness) in accordance with indications for the use of acrylic resin for prosthodontics applications, considering the 3D print processing methods (printed PMMA), milled PMMA, and the traditional method (heat-curing PMMA).

#### 3.1.1. Traditional Method

Discoidal samples with a diameter of 12 mm and a thickness of 3 mm were obtained using the traditional dental laboratory method. The samples were made of heat-curing acrylic resin (Superpont C + B acrylic resin, Spofadental, Jičín, Czechia) [32]. This material is delivered with the following components: polymer powder and monomer liquid. The acrylic resin paste was placed in metal molds to obtain the discoidal shape, applying pressure to remove any air bubbles and ensure the uniform distribution of the material in the mold. The molds filled with acrylic resin were subjected to the polymerization regime (100 °C, 3 barr atm., 40 min.). The curing was carried out according to the manufacturer’s instructions, ensuring that the resin achieves the required hardness and stability. After polymerization, the samples are removed and allowed to cool.

#### 3.1.2. Subtractive Method (Milling)

The same shape and size samples from PMMA material were milled from industrially prefabricated CORitech Temp PMMA Disc, on the CORiTEC 350i machine (manufactured by IMES I-CORE GMBH, Hessen, Germany) [33].

#### 3.1.3. Additive Method (3D Printing)

Additional discoidal samples of identical dimensions were fabricated using the digital light processing (DLP) technique and liquid acrylic resin, specific for temporary crowns (VarseoSmile Temp, Bego, Germany) [35] and splints (FotoDent splint (LCD), Dreve Dentamid GmbH, Unna, Germany) [36]. The 3D printer used was Planmeca Creo C5 (Planmeca, Finland). Samples with well-defined morphological and structural characteristics were obtained, essential for subsequent evaluations.

### 3.2. Indentation Tests

The nanomechanical properties (Young’s modulus (E), hardness (H), and stiffness (N)) of all acrylic resin samples were investigated by nanoindentation, using the Nano Indenter G200 system (Keysight Technologies, Santa Rosa, CA, USA). The specimens were fixed on the sample holder, and the indentation tests were carried out at room temperature using a triangular pyramid Berkovich diamond indenter with a 100 nm radius. The express test to a displacement method was applied, performing 400 indents (20 × 20) at 50 μm distance from each other (to prevent interactions between indentations) in 3 different points on each discoidal sample. Indentations were set to a maximum penetration depth of 500 nm to mitigate surface effects, and meanwhile, to avoid damage to the specimen and substrate influence [37,38]. The results for each nanomechanical parameter represent the average values over all valid indents performed at each point on the corresponding sample.

### 3.3. Micro-CT Analysis

For the quantitative morphological characterization of porosity from acrylic resin samples obtained by 3D printing and CAD/CAM milling, micro-computed tomography was used. Micro-CT analysis was performed on a Bruker SkyScan 1272 microCT scanner (Bruker, Kontich, Belgium), which uses conical radiation to scan samples at a high resolution of 4904 × 3280 pixels. The scan parameters included a voltage of 45 kV and an emission current of 150 μA, ensuring adequate penetration and optimal image quality. The scan settings are: pixel size 0.7 μm, average frames: for each projection, angular pitch: 0.2°, exposure time: 600 ms per frame, full 360° rotation, resulting in a total scan time of approximately 2 h and 30 min, with over 3000 sections obtained for each sample [39].

The raw images obtained from the scan were later reconstructed using the CTR econ software (version 1.7.1.6). This process involves transforming 2D projections into a 3D volume by applying tomographic reconstruction algorithms, such as filtered back projection.

**Figure 1:**
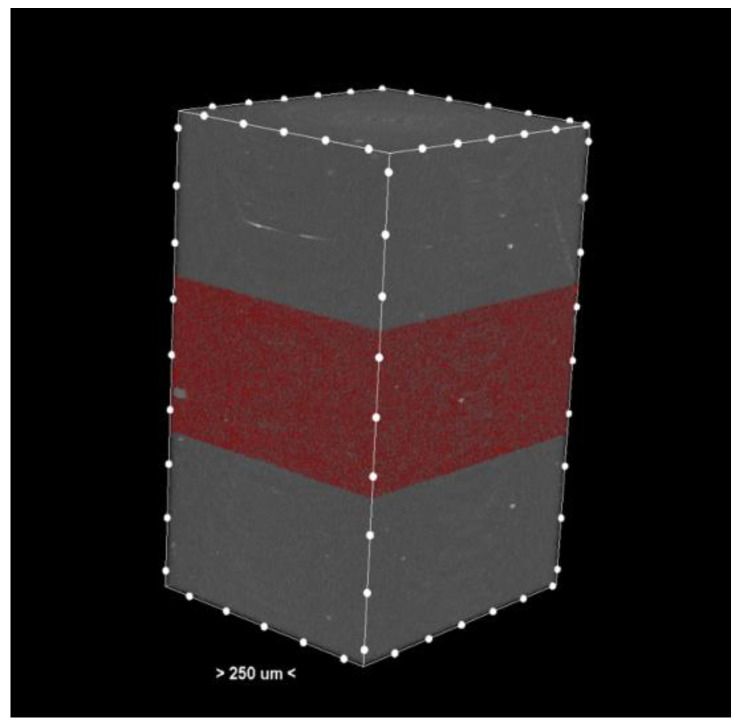
Cross-section—3D reconstruction of the sample analyzed by micro-CT. The pores or the materials were marked red in the middle section.

The resulting cross-sections (Figure 1) provide a detailed representation of the internal structures of the samples.

For the visualization of 3D objects, cross-sections were used in the CTVox software (version 3.3. or 1403). This software allows for the manipulation and exploration of the 3D volumes obtained, providing a clear perspective on the morphological characteristics of the samples.

**Figure 2:**
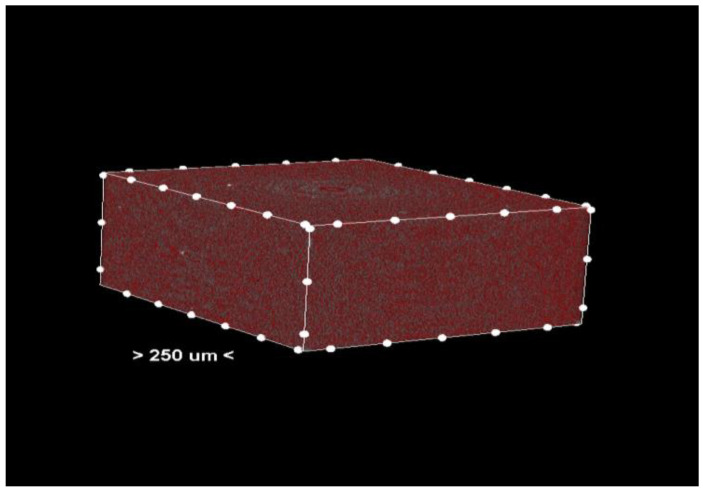
Center section—pores (colored red) in the material of the analyzed sample.

**Figure 3:**
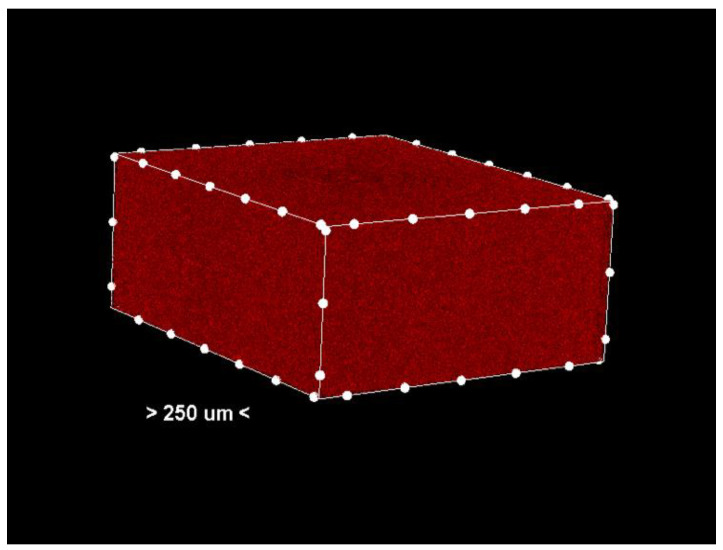
Center section—only the pores (colored red) in the analyzed sample. Non-porous material has been removed from the generated images.

**Figure 4:**
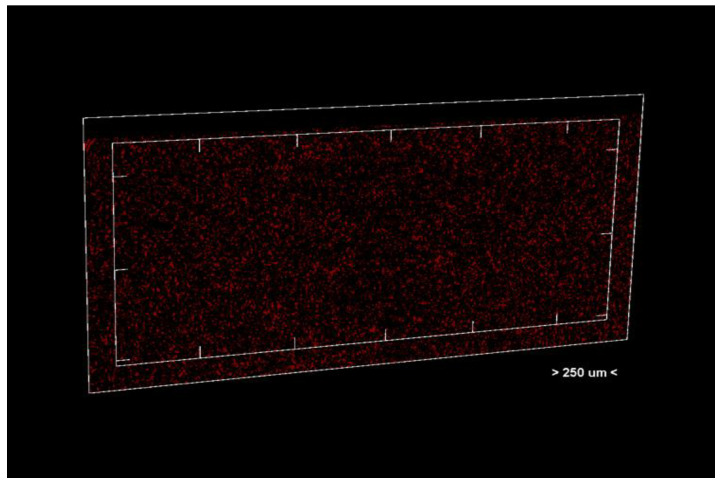
Unit section of the analyzed material.

Porosity analysis was performed with the CTAn software, version 1.18.4.0. This software allows the identification and quantification of pores in the sample structure by marking them in red in the center section for easy visualization (Figure 2 and Figure 3, center section, and Figure 4, unit section).

The segmentation process separates the pores from the solid material based on density differences. The identified pores are marked in red in the central sections of the samples (Figure 3) to facilitate visual and quantitative analysis. The software calculates specific parameters such as pore volume, size distribution, and pore density, providing detailed information about the internal structure of the samples.

### 3.4. Statistical Analysis

The experimental data were statistically processed using the GraphPad Prism software, version 8.0.1. The statistical method applied was two-way ANOVA, and statistical significance was determined for values of *p* < 0.05. The adoption of a *p*-value < 0.05 as a significance threshold ensured that the observed differences were statistically relevant, thus providing confidence in the validity of the study’s conclusions. The results are presented as mean ± standard deviation, where the mean represents the central value of the data set, and the standard deviation indicates the variability of the data around the mean. Number of replicates, *n* = 3. Each experiment was performed three times, and the combined results from these replicates were used for statistical analysis. This ensures the reproducibility and reliability of the results.

## 4. Results

### 4.1. Results of Nanomechanical Investigations

Table 1, Table 2, Table 3 and Table 4 present the results of nanomechanical evaluation (Young’s modulus, hardness, and rigidity) for the samples prepared via different methods.

**Figure 5:**
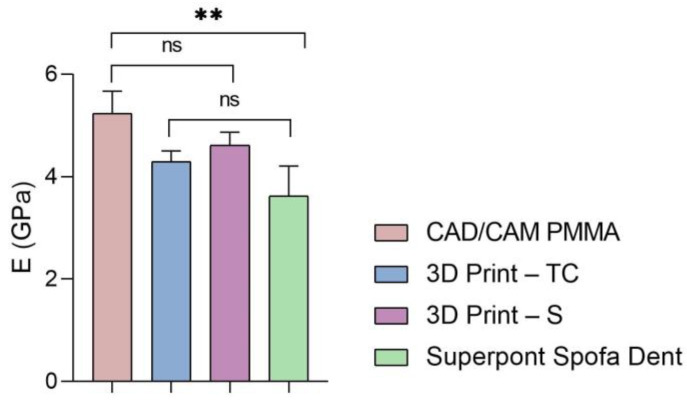
Elastic modulus of all investigated samples (statistical significance: ns > 0.5; ** *p* < 0.005).

**Figure 6:**
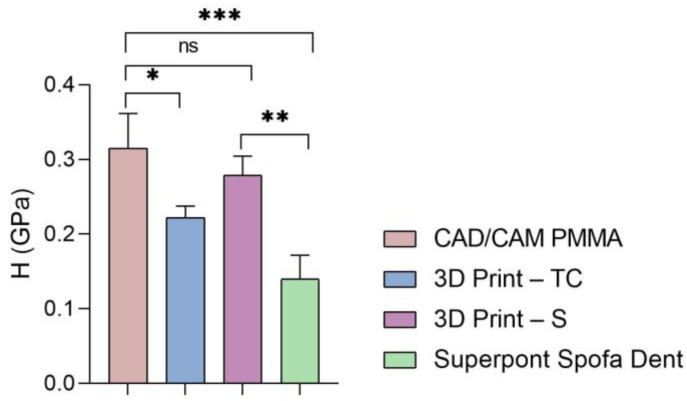
Hardness of all tested specimens (statistical significance: ns > 0.5; * *p* < 0.05; ** *p* < 0.005; *** *p* < 0.001).

**Figure 7:**
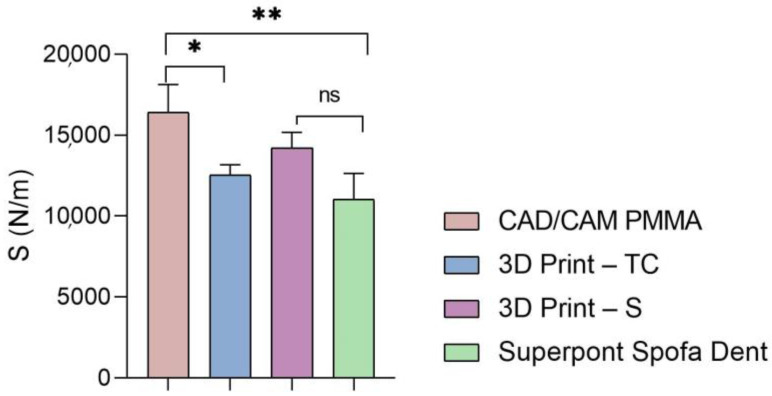
Stiffness of analyzed samples (statistical significance: ns > 0.5; * *p* < 0.05; ** *p* < 0.005).

Furthermore, the global values of elastic modulus, hardness, and stiffness measured for each sample are presented in Figure 5, Figure 6 and Figure 7.

According to the results (Figure 5 and Table 1, Table 2, Table 3 and Table 4), the highest values of elastic modulus (E) are recorded for the restorative dental material fabricated by CAD/CAM milling technology (CAD/CAM PMMA) and are in the range of 5.181–5.692 GPa, followed by samples fabricated through additive manufacturing: 3D print–S splints with the E in the range of 4.445–4.908 GPa, respectively 3D Print–TC temporary crowns (E between 4.125–4.524 GPa). Among the investigated specimens, the lowest elastic modulus is registered for the Superpont Spofa Dent sample obtained by the traditional method, with an E value between 2.984 and 4.121 GPa. Overall, the CAD/CAM PMMA material exhibits the lowest elasticity, and consequently the highest rigidity and resistance to deformation, in contrast to Superpont Spofa Dent restorative dental material, which is characterized by improved flexibility and reduced rigidity.

Then, a similar trend was registered when the hardness and stiffness of fabricated samples were evaluated (Figure 6 and Figure 7 and Table 1, Table 2, Table 3 and Table 4). The milled CAD/CAM PMMA material shows the highest values of hardness and stiffness, ranging between 0.27 and 0.362 GPa, respectively, 12,537 N/m, while the lowest values are registered for specimens fabricated through traditional thermos-baro polymerization (Superpont Spofa Den), in accordance with elastic modulus results. Therefore, milled CAD/CAM PMMA material will require a higher load to reach the penetration depth (~1.8 mN) as compared to Superpont Spofa Dent material, for which an approximately two-fold decrease in load (~0.8 mN) was needed to reach the targeted indentation depth of 500 nm.

### 4.2. Porosity Evaluation by Micro-CT

**Figure 8:**
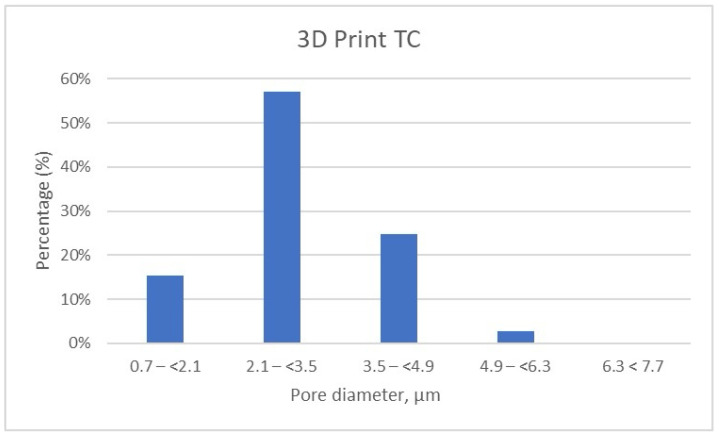
Porosity Distribution of 3D Print Temporary Crown Samples.

**Figure 9:**
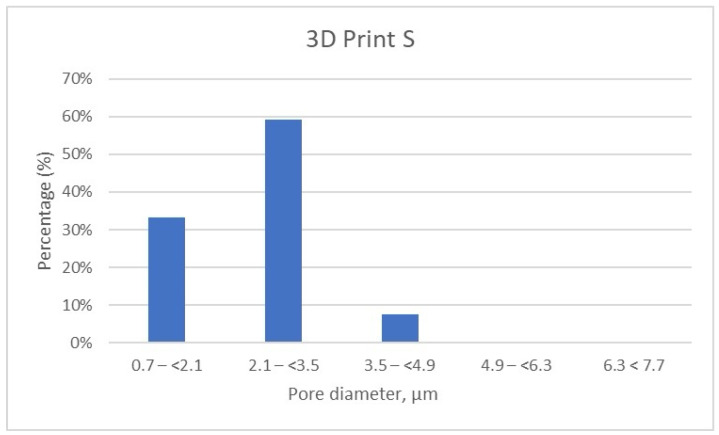
Porosity distribution of 3D Print–Splint samples.

**Figure 10:**
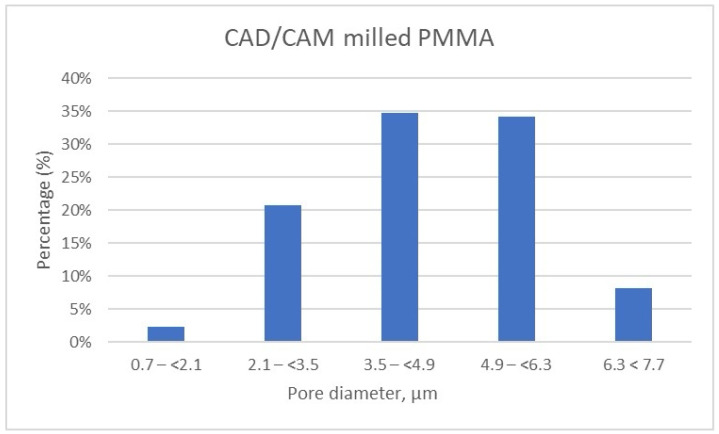
Porosity distribution of CAD/CAM milled samples.

The detailed results of pore size distribution are centralized in Table 5, Table 6 and Table 7, while graphical representations of pore distribution for each sample are illustrated in Figure 8, Figure 9 and Figure 10.

It can be noted that the specimens fabricated through the additive manufacturing method are characterized by porosity with pore sizes primarily in the range of 2.1 to 3.5 μm, accounting for more than 50% of the total porosity in the 3D Print-TC temporary crowns (Table 5, Figure 8). Specifically, this dental material predominantly has pores smaller than 3.5 μm, with 15.41% of the pores in the range 0.7–(<2.1) μm, 57% of the pores being found in the range 2.1–(<3.5) μm and about 25% in the range 3.5–(<4.9) μm.

Further, the results show that in the 3D Print S—Splint material, over 90% of the pores fall within the 0.7–(<2.1) μm (33.19%) and almost 60% of pores are found in the range of 2.1–(<3.5) μm (Table 6, Figure 9). Very few pores, about 7%, are found in the range of 3.5–(<4.9) μm.

Meanwhile, the pore size distribution of CAD/CAM-fabricated PMMA restorative dental material is wider as compared to its 3D printed counterparts; approximately 70% of the pores have diameters ranging between 3.5 μm and 6.3 μm, and a very small amount of pores are smaller than 2.1 μm (less than 2.5%) (Table 7, Figure 10). Moreover, the porosity analysis indicates that up to 10% of the total pores exceed 6.3 μm in size, whereas in the 3D printed samples, such larger pores are present only in negligible quantities.

**Figure 11:**
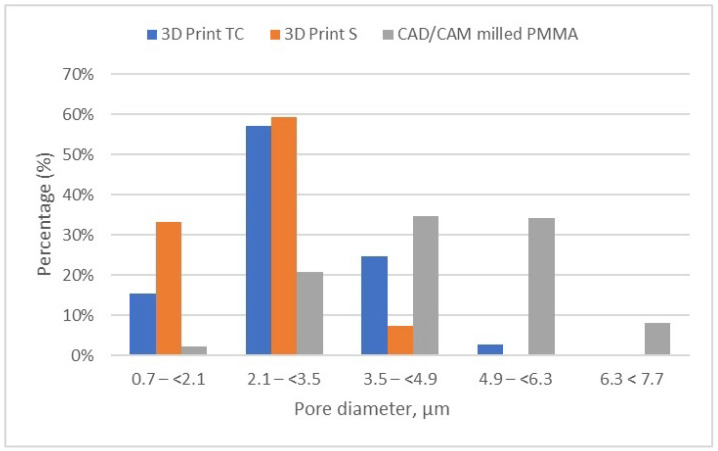
Porosity distribution of the evaluated samples (3D print TC, 3D print S, and milled PMMA).

Figure 11 provides a comparative analysis of the pore size and distribution across all samples investigated. The data indicates that materials fabricated via 3D printing exhibit smaller pore sizes compared to those produced through CAD/CAM milling. These differences in porosity can significantly influence the overall performance of the materials, potentially impacting properties such as strength, durability, and biological integration.

It is well known that detailed pore analysis is a crucial aspect of materials characterization that involves examining and quantifying the internal pore structure within a material at the microscopic level. This process provides comprehensive insights into parameters such as pore size distribution, pore volume, porosity, shape, and connectivity, all of which significantly influence the mechanical features of the material, permeability, and overall performance.

The porosity analysis outcomes for the investigated materials are detailed in Table 8, while Table 9 summarizes the overall results and presents the average values, offering a comprehensive overview of the porosity characteristics across the samples studied.

The results indicated a decreasing trend in the percentage of total porosity when comparing 3D printed restorative materials to milled CAD/CAM specimens. So, 3D Print–TC temporary crowns are characterized by a higher percent of total porosity (~22.93%), among which ~22.69% is represented by open porosity and ~0.31% is closed porosity. Next, the total porosity of 3D Print–S splint is approximately three-fold lower (~8.94%) than that of 3D Print–TC, and its porosity is mainly composed of closed pores (~6.62%) rather than open pores (~2.48%). The lowest total porosity value is registered for milled CAD/CAM PMMA restorative dental material (~5.72%), but this porosity consists exclusively of open pores.

## 5. Discussion

**Nanomechanical characteristics**

It is well-known that the nanomechanical characteristics of materials with potential application in dental tissue restoration represent crucial factors in defining their clinical efficiency and application. In this respect, nanoindentation represents a powerful approach in the accurate evaluation of mechanical performances of dental materials, owing to its high spatial resolution, simplicity, and ability to make nano/microscale measurements. Elastic modulus is an intrinsic property of a material, unlike hardness, which is an engineering property [40].

Regarding the modulus of elasticity of the samples investigated, it can be noted that the results indicate a higher structural strength of CAD/CAM milled PMMA dental restorative material, which may suggest an increased resistance to deformation under applied loads. PMMA is often used for temporary dental restorations due to a combination of sufficient strength and the ability to be easily machined. This mechanical characteristic is important for applications where structural stability is central. The 3D printed methacrylate resins for splints (3D print-S), although with lower values than those for the traditional method, still exhibit significant structural strength. Dental splints must be rigid enough to provide adequate support and protection, but at the same time, they can exhibit slightly greater flexibility compared to more rigid materials. The structural strength of 3D temporary crowns is in a similar range to that of methacrylate resins for splints, which is appreciated as adequate strength for temporary use in dentistry and sufficient flexibility. Superpont Spofa Dent specimen obtained by the traditional method demonstrates the lowest modulus of Young’s elasticity of all those mentioned in the given context. In general, higher values of Young’s modulus of elasticity indicate less flexibility of the material and a greater ability to return to its original shape after loading.

Higher resistance to deformation and improved structural stability are recorded for the samples fabricated by CAD/CAM milling technology, presenting the maximum modulus of elasticity.

These results are consistent with those reported by Valenti et al. [1], who demonstrated superior stiffness in PMMA materials processed via subtractive techniques compared to those produced using traditional or additive methods. Similarly, those restorations manufactured through CAD/CAM milling exhibit enhanced structural stability due to the homogeneous microstructure and high polymerization degree obtained under controlled industrial conditions [22].

For splints, 3D-printed methacrylate resins offer a compromise between strength and flexibility with lower but clinically acceptable values. Among the tested samples, the specimen obtained by the conventional approach presents the lowest mechanical characteristics.

Concerning the hardness of the investigated samples, it can be mentioned that the milled PMMA-based CAD/CAM restorative material indicates a high hardness.

These elevated hardness values align with other authors’ findings, who observed that industrially fabricated composite materials exhibit increased durability due to optimal polymerization and the absence of interconnected porosity. For 3D printed methacrylate resins, the moderate hardness values observed are in accordance with those reported in the literature, where it is shown that while such materials display clinically acceptable performance, their lower hardness limits their use in restorations subjected to significant masticatory stress [2,24].

This material is suitable for applications where strength and durability are essential, such as temporary or even permanent dental crowns and various dental devices. In general, PMMA is widely employed for temporary restorations due to its adequate strength, although its durability remains comparatively lower than that of materials specifically designed for permanent applications. The 3D printed methacrylate resins for splints have lower values than those obtained by the traditional method, but still indicate significant hardness. Dental splints must be hard enough to withstand wear and deformation during use. The hardness of 3D temporary crowns has values in a similar range to that of methacrylate resins for splints, reflecting adequate strength for their temporary use in dentistry. Superpont Spofa Dent sample obtained by the traditional method exhibits the lowest hardness of all those mentioned in the given context. In general, higher hardness values indicate greater resistance to deformation and wear. Differences in hardness can influence clinical and design decisions in dentistry, ensuring that the materials selected are appropriate for the specific requirements of each application.

As for the stiffness of the investigated samples, it can be noted that the sample obtained by CAD/CAM milling technology indicates a high stiffness. This suggests high resistance to deformation and excellent structural stability, which are essential in applications that require durable and resistant materials. The 3D printed methacrylate resins for splints have slightly lower values than those for the traditional method, but still indicate significant stiffness. Dental splints must be rigid enough to provide adequate support and protection, but they can have greater flexibility than more rigid materials. The stiffness of the temporary crowns is lower than that of methacrylate resins for splints and that of the traditional method. These materials are often used temporarily in dentistry, where short-term resistance to deformation may be sufficient. The dental restorative specimen fabricated by the traditional method displays the lowest stiffness of all those mentioned in the given context.

These observations are in agreement with other findings [34], which reported increased internal porosity in 3D printed dental restorations, attributed to the layered fabrication process and potential for incomplete polymerization. In contrast, found that industrially milled PMMA materials exhibit a denser structure and more uniform pore distribution, resulting in superior mechanical properties and greater clinical reliability [26].

In conclusion, higher stiffness values indicate a greater ability of the material to resist deformation and maintain shape under loads, which is important in permanent dental applications. Differences in stiffness can influence the selection of materials and the design of dental restorations, ensuring that materials with appropriate mechanical properties are chosen for each specific application, temporarily or long-term.

**Porosity characteristics**

It is well reported that porosity represents a key factor in dental restorative applications, as it directly impacts the mechanical integrity, biocompatibility, and long-term clinical performance of restorative materials [12,41]. All samples analyzed show both closed and open porosity, in different proportions, depending on the composition and/or manufacturing method. The 3D Print–TC acrylic resin obtained by 3D printing can be useful for temporary crowns, but should be carefully evaluated to ensure that it does not compromise performance in the medium and long term. This pore distribution shows that the three-dimensional printed material has a predominantly dense structure with small and medium pores, which can be beneficial for use in temporary crowns, as it can provide an optimal combination of strength and durability. At the same time, the resin obtained by CAD/CAM milling of the PMMA disk is preferable for medium-term solutions and high-precision applications, given the density and uniformity of porosity.

The distribution of open and closed porosity observed in this study is consistent with the findings of Revilla-León et al. [25], who demonstrated that 3D printed photopolymerized materials often exhibit predominantly open porosity, which can affect fluid absorption and adhesion. In a more recent study, Dimitrova et al. [13] investigated the water sorption and solubility of various dental base materials, including 3D-printed resins. The authors suggested that the inherent open porosity of 3D-printed photopolymerized dental materials plays a critical role in influencing their fluid absorption capacity and the adhesion properties of restorative interfaces, in agreement with previous findings.

Conversely, the high proportion of closed porosity identified in splint materials may offer mechanical advantages by improving dimensional stability and limiting fluid permeability in the oral environment [34].

Samples with predominantly open porosity are 3D Print–TC and CAD/CAM PMMA (more than 99% of the total porosity is open), while 3D Print–C mostly exhibits closed pores (almost 75% of the total porosity is closed). Open pores are defined as those connected to the external surface of the material, potentially influencing properties such as permeability and adhesion. In the case of 3D Print–TC, the presence of high open porosity might be acceptable for temporary applications; however, for CAD/CAM PMMA, this characteristic could be less desirable, considering that milled materials are generally favored for their homogeneity and structural density. The 3D Printed S sample (3D Print Acrylic Resin for Splints) exhibits approximately 75% closed porosity, reflecting a microstructure in which a substantial portion of pores lack surface connection. Although closed pores pose fewer concerns regarding infiltration or fluid permeability, they may still affect mechanical aspects such as flexibility and the material’s stress response. This structural feature may be particularly advantageous in the case of dental splints, where closed porosity helps maintain a stable internal environment and limits fluid infiltration.

**Limitations of this Study**

This study presents several limitations that must be acknowledged. First, although the selected acrylic resins represent commonly used materials in current dental practice, they have different clinical indications—such as temporary crowns and splints—which may inherently influence their mechanical behavior and internal structure. While our aim was to evaluate the influence of processing method across various clinical categories, the inclusion of materials with distinct intended uses may affect the direct comparability of results.

Second, only a limited number of commercial products were analyzed for each fabrication technique, which may not fully capture the variability across available brands or material compositions. Future studies should include a broader range of materials with matching clinical indications to allow for more direct comparisons.

This study was limited to a specific selection of commercially available acrylic resins representative of three processing techniques (traditional, 3D printing, and CAD/CAM milling). The results are based on a limited number of samples and materials, which may not fully capture the variability across brands or formulations. In addition, only nano-mechanical properties and porosity were analyzed; other relevant aspects such as surface roughness, wear resistance, or in vivo behavior were not evaluated.

Moreover, the scope of this investigation was restricted to nano-mechanical properties (elastic modulus, hardness, and stiffness) and microstructural porosity. Other clinically relevant characteristics, such as wear resistance, surface roughness, color stability, water absorption, and bonding behavior, were not evaluated. These factors are also essential for the comprehensive assessment of temporary prosthodontic materials.

In addition, in vivo factors such as thermal cycling, masticatory forces, and exposure to the oral environment were not simulated. Therefore, the extrapolation of in vitro results to clinical performance should be performed with caution. Future studies should expand the sample size, include more material types, and incorporate additional functional tests.

## 6. Conclusions

The processing method significantly influences both the nano-mechanical performance and internal porosity of acrylic resins used for dental applications. The clinical performance of dental materials is determined not only by their physical and chemical characteristics but also by mechanical properties such as elastic modulus, hardness, and stiffness, along with microstructural aspects like porosity, which may significantly influence behavior under functional loading.

In this study, the use of advanced characterization techniques such as nanoindentation and micro-computed tomography allowed for a comprehensive evaluation of nanomechanical properties and internal porosity across acrylic resin materials processed by three different methods: traditional heat-curing, additive 3D printing, and subtractive CAD/CAM milling.

Among the tested samples, the CAD/CAM PMMA-based material demonstrated the highest values for modulus of elasticity, hardness, and stiffness, suggesting a high degree of structural stability and resistance to deformation, thus making it suitable for medium-term and precision-demanding clinical applications where rigidity and dimensional stability are critical. The 3D printed methacrylate resins for splints exhibited considerable mechanical performance, balancing strength and flexibility, while materials intended for temporary crowns showed intermediate values that are adequate for short-term restorations. The splint-specific resin showed higher stiffness and closed porosity compared to the temporary crown resin, which exhibited increased flexibility and predominantly open porosity. All these characteristics may influence clinical decisions, which are dependent on the specific functional requirements: load bearing and flexibility, short-term use, and extended use. By contrast, the traditionally polymerized Superpont material recorded the lowest mechanical values across all parameters, indicating limited structural strength and suggesting its suitability mainly for provisional uses where lower mechanical demands are acceptable. Having low values for mechanical parameters, the heat-cured resin is indicated for applications that do not require significant structural durability. It also remains relevant due to cost-effectiveness for short-term restorations and technological familiarity.

Furthermore, the detailed micro-CT analysis revealed significant differences in porosity characteristics among the studied groups. The 3D printed materials exhibited increased total porosity and a wider pore size distribution, with the temporary crown resin showing over 22% total porosity, primarily composed of open pores. In comparison, CAD/CAM PMMA showed the lowest porosity (approximately 5.7%), with a more compact and uniform internal structure. The splint resin displayed a distinct profile, with total porosity around 9%, characterized predominantly by closed pores, which may contribute to better dimensional stability and reduced fluid absorption. The observed variation in open and closed pore proportions is directly linked to the processing method and material formulation. In the case of CAD/CAM and 3D printed crown samples, the high share of open porosity may influence permeability and surface interactions, whereas the closed porosity in splint samples could enhance barrier functions and internal cohesion. Moreover, differences in mechanical behavior can be attributed not only to the type of processing technique but also to polymerization efficiency, post-processing treatments such as finishing and polishing, and the degree of conversion, all of which influence the final structure and material homogeneity. In addition, the recent literature highlights the increasing role of artificial intelligence in digital prosthodontics, with an impact on material selection, diagnostic accuracy, and workflow automation [42]. Consequently, the optimal selection of a processing method should be guided by the specific clinical indications, considering both mechanical demands and the implications of porosity on aesthetic, functional, and biological performance. The clinical relevance of these findings must be taken into consideration in the context of the material’s intended use. This study focused on comparing processing techniques, and it is important to underline that the materials developed for different applications (e.g., crowns vs. splints) are optimized differently by manufacturers. Therefore, future research should take into consideration comparing materials and testing them to simulate clinical conditions more accurately.

In conclusion, the best selection of a processing method should not rely solely on material strength or manufacturing convenience, but also consider the interplay between mechanical demands, porosity characteristics, and the clinical context in which the material will be used.

## Figures and Tables

**Table 1:** Nanomechanical characteristics of milled PMMA.

Test	Surface Displacement	Sample Loading	Elasticity (Y Module)	Hardness	Stiffness
	nm	Mn	GPa	GPa	N/m
1	533.5	1.7	5181	0.314	16,043
2	528.0	1.5	4828	0.270	14,946
3	559.8	2.1	5692	0.362	18,284
Mean	540.4	1.8	5233	0.315	16,424
Std. Dev.	17.0	0.3	0.434	0.046	1701

**Table 2:** Nanomechanical characteristics of 3D Print–TC.

Test	Surface Displacement	Sample Loading	Elasticity (Y-Module)	Hardness	Stiffness
	nm	Mn	GPa	GPa	N/m
1	485.1	1.0	4125	0.205	11,910
2	502.6	1.2	4233	0.235	12,514
3	490.7	1.1	4524	0.226	13,188
Mean	492.8	1.1	4294	0.222	12,537
Std. Dev.	8.9	0.1	0.206	0.015	640

**Table 3:** Nanomechanical characteristics of 3D Print–G.

Test	Surface Displacement	Sample Loading	Elasticity (Y-Module)	Hardness	Stiffness
	nm	Mn	GPa	GPa	N/m
1	540.2	1.7	4908	0.308	15,327
2	526.2	1.4	4445	0.261	13,618
3	527.8	1.4	4486	0.268	13,714
Mean	531.4	1.5	4613	0.279	14,220
Std. Dev.	7.7	0.2	0.256	0.025	960

**Table 4:** Nanomechanical characteristics of Superpont Spofa Dent.

Test	Surface Displacement	Sample Loading	Elasticity (Y-Module)	Hardness	Stiffness
	nm	Mn	GPa	GPa	N/m
1	523.6	0.6	2984	0.111	9194
2	498.7	0.9	4121	0.174	12,200
3	512.1	0.8	3766	0.135	11,693
Mean	511.2	0.8	3552	0.142	10,697
Std. Dev.	17.6	0.2	0.804	0.044	2125

**Table 5:** Micro-CT results for the 3D Print TC sample. Distribution of pores according to size by intervals.

3D Print TC—Temporary Crowns					
Sections 150–1149		Sections 1150–2149	Sections 2150–3149	Intercede	Std. Dev.
Interval, μm	% in range	% in range	% in range	% in range	
0.7–<2.1	14.39	15.31	16.55	15.41	0.89
2.1–<3.5	56.22	57.11	57.74	57.03	0.62
3.5–<4.9	26.21	24.84	23.24	24.76	1.22
4.9–<6.3	3.15	2.71	2.39	2.75	0.31
6.3 <	0.03	0.03	0.09	0.05	0.03

**Table 6:** Micro-CT results for the 3D Print S sample. Distribution of pores according to size by intervals.

3D Print S—Splint					
	Sections 150–1149	Sections 1150–2149	Sections 2150–3149	Intercede	Std. Dev.
Pore diameter, μm	% in range	% in range	% in range	% in range	
0.7–<2.1	34.31	32.28	32.98	33.19	0.84
2.1–<3.5	58.81	59.77	59.31	59.30	0.39
3.5–<4.9	6.77	7.80	7.59	7.39	0.44
4.9–<6.3	0.10	0.14	0.12	0.12	0.02
6.3 < 7.7		0.00		0.00	0.00

**Table 7:** Micro-CT results for CAD/CAM PMMA milled sample. Distribution of pores according to size by intervals.

CAD/CAM Milled PMMA					
	Sections 150–1149	Sections 1150–2149	Sections 2150–3149	Intercede	Std. Dev.
Pore diameter, μm	% in range	% in range	% in range	% in range	
0.7–<2.1	2.33	2.38	2.13	2.28	0.11
2.1–<3.5	21.04	21.41	19.60	20.68	0.78
3.5–<4.9	34.88	35.30	34.12	34.77	0.49
4.9–<6.3	33.87	33.42	35.11	34.13	0.71
6.3 < 7.7	7.88	7.49	9.04	8.14	0.66

**Table 8:** Pore weight and porosity by pore type on the analyzed sections.

Sample	Section	Total Porosity, %	Open Porosity, %	Closed Porosity, %
3D Print–TC	150–1149	24.644	24.476	0.22195
	1150–2149	23.403	23.198	0.26769
	2150–3149	20.75	20.403	0.4368
3D Print–S	150–1149	8.4264	1.7616	6.7842
	1150–2149	9.5977	3.565	6.2557
	2150–3149	8.8072	2.1191	6.8329
milled PMMA	150–1149	5.7364	5.7363	0.0038021
	1150–2149	5.6784	5.6783	0.0041061
	2150–3149	5.7648	5.7647	0.0036571

**Table 9:** Pore size shares on average and average porosity, depending on pore type.

Sample	Average Total Porosity, %	Std. Dev.	Average Open Porosity, %	Std. Dev.	Average Closed Porosity, %	Std. Dev.
3D Print–TC	22.93	1.62	22.69	1.70	0.31	0.09
3D Print–S	8.94	0.49	2.48	0.78	6.62	0.26
milled PMMA	5.727	0.36	5.72	0.36	0.00	0.00

## Data Availability

The original contributions presented in this study are included in the article; further inquiries can be directed to the corresponding author.
